# Coxsackievirus B3 Inhibits Antigen Presentation *In Vivo*, Exerting a Profound and Selective Effect on the MHC Class I Pathway

**DOI:** 10.1371/journal.ppat.1000618

**Published:** 2009-10-16

**Authors:** Christopher C. Kemball, Stephanie Harkins, Jason K. Whitmire, Claudia T. Flynn, Ralph Feuer, J. Lindsay Whitton

**Affiliations:** 1 Department of Immunology and Microbial Science, SP30-2110, The Scripps Research Institute, La Jolla, California, United States of America; 2 Department of Biology, San Diego State University, San Diego, California, United States of America; University of Pennsylvania School of Medicine, United States of America

## Abstract

Many viruses encode proteins whose major function is to evade or disable the host T cell response. Nevertheless, most viruses are readily detected by host T cells, and induce relatively strong T cell responses. Herein, we employ transgenic CD4^+^ and CD8^+^ T cells as sensors to evaluate *in vitro* and *in vivo* antigen presentation by coxsackievirus B3 (CVB3), and we show that this virus almost completely inhibits antigen presentation *via* the MHC class I pathway, thereby evading CD8^+^ T cell immunity. In contrast, the presentation of CVB3-encoded MHC class II epitopes is relatively unencumbered, and CVB3 induces *in vivo* CD4^+^ T cell responses that are, by several criteria, phenotypically normal. The cells display an effector phenotype and mature into multi-functional CVB3-specific memory CD4^+^ T cells that expand dramatically following challenge infection and rapidly differentiate into secondary effector cells capable of secreting multiple cytokines. Our findings have implications for the efficiency of antigen cross-presentation during coxsackievirus infection.

## Author Summary

Many viruses—for example, large DNA viruses like smallpox virus and herpesviruses—encode several proteins whose major function is to combat the host's immune response, but these proteins usually battle in vain; in general, the mammalian immune system is sufficiently accomplished to penetrate this viral armor, allowing the infected animal to mount an immune response that can eradicate—or, at least, suppress—the infectious agent. Here, we show that coxsackievirus, a small RNA virus, carries a far more powerful punch than its larger DNA cousins; it almost entirely evades detection by host CD8^+^ T cells, which usually are one of the key components of an antiviral immune response. How does the virus achieve such success? Normally, when a virus infects a cell, certain host proteins capture small fragments of the virus and display them on the cell's surface, allowing them to be detected by the host immune system—usually, by cells called CD8^+^ T cells. We show here that coxsackievirus very effectively prevents these “flags” from reaching the cell surface in a form that can trigger naïve T cells to respond; in effect, the virus renders the cell “invisible” to CD8^+^ T cells, creating a cocoon in which the virus can multiply undisturbed by host immunity.

## Introduction

Most virus infections are potent inducers of T cell activation and expansion, and viruses employ several strategies to elude these T cell responses. Some viruses – in particular, RNA viruses – evade T cells by changing their amino-acid composition, while others – exemplified by the herpesvirus family – establish a latent infection, in which viral antigen production is terminated, rendering the infected cell invisible to T cells. Still more viruses – for example, the poxviruses – encode factors that inhibit or misdirect the effector functions of T cells. Despite these immunoevasion strategies, in the great majority of acute virus infections that have been studied T cell responses are readily detected directly *ex vivo*. This is not to say that the evasive factors encoded by these viruses have no *in vivo* effect, but rather the virus' ability to evade host T cell responses is far from complete.

Our laboratory, and others, have investigated the diseases caused by type B coxsackieviruses (CVB), and the underlying pathogenic mechanisms. CVB are important human pathogens that belong to the picornavirus family and enterovirus genus. A considerable proportion of CVB infections trigger severe acute and chronic diseases and cause morbidity and mortality, particularly in infants, young children, and immunocompromised individuals [1]–[3]; CVB are the most common cause of infectious myocarditis, a serious disease that can lead to dilated cardiomyopathy and cardiac failure [4]–[6], and also frequently induce pancreatitis and aseptic meningitis [1], [7]–[10]. One goal of our early studies was to map T cell epitopes in the virus, but progress was limited because the virus infection induced remarkably weak T cell responses [11],[12]. To enhance our ability to detect and analyze CVB-specific T cell responses, we generated a recombinant CVB3 (rCVB) that expresses well-characterized CD8^+^ and CD4^+^ T cell epitopes derived from lymphocytic choriomeningitis virus (LCMV). We have shown that this virus failed to induce strong endogenous primary CD4^+^ and CD8^+^ T cell responses *in vivo*, but could be recognized by LCMV-specific memory cells, and we speculated that CVB3 might reduce presentation of viral antigens to a level that is sufficient to trigger memory, but not naïve, T cells [12]. In this study, we have used epitope-specific transgenic CD4^+^ and CD8^+^ T cells, together with additional rCVB3, to better evaluate CVB3-specific T cell responses, and to assess the virus' ability to inhibit the presentation of MHC class I and class II epitopes *in vitro* and *in vivo*. With this enhanced detection system we show that CVB3 induces essentially no detectable *in vivo* primary CD8^+^ T cell response. This near-complete evasion of CD8^+^ T cell immunity does not result from the virus' destroying or paralyzing epitope-specific CD8^+^ T cells; nor does the virus induce a global suppression of the animal's ability to mount strong T cell responses. Using the transgenic T cells as sensors of *in vitro* and *in vivo* antigen presentation, we conclude that CVB3 profoundly inhibits the MHC class I antigen presentation pathway, providing a plausible explanation for the weakness of the primary CD8^+^ T cell response. In contrast, MHC class II presentation is relatively uninterrupted, and CVB3 induces both primary and memory CD4^+^ T cell responses. In addition, we describe the quantity and quality of memory CD4^+^ T cells that are induced by CVB3 infection. Thus, we show that CVB3 selectively inhibits the MHC class I pathway; our findings also have implications for the *in vivo* efficiency of cross-priming during CVB3 infection.

## Results

### Minimal *in vivo* activation of endogenous CD4^+^ and CD8^+^ T cells following infection with wildtype or recombinant CVB3

**Figure 1:**
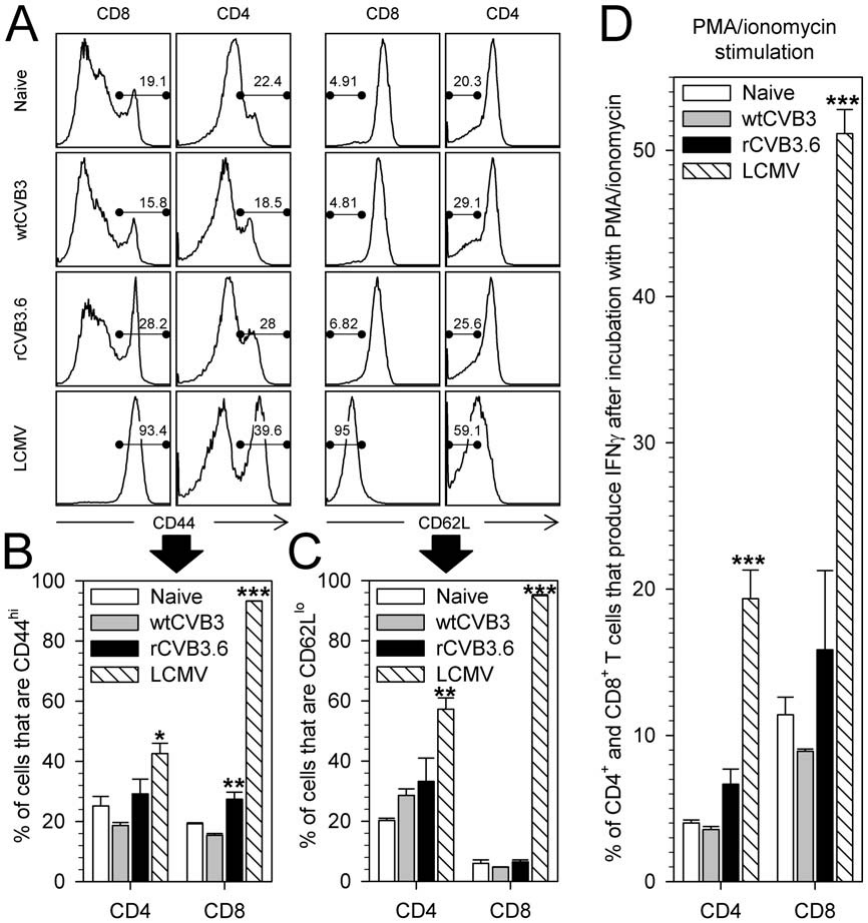
Minimal T cell activation following infection with wild-type or recombinant CVB3. Mice were infected with wtCVB3, rCVB3.6, or LCMV as a positive control, and the extent of T cell activation was analyzed on day 8. (A) CD44 and CD62L expression profiles of CD8^+^ or CD4^+^ splenocytes were compared for naïve and virus-infected mice. Representative histograms are gated on CD8^+^ or CD4^+^ cells, and the numbers indicate the proportion of cells that are CD44^hi^ or CD62L^lo^. The frequency of CD44^hi^ (B) or CD62L^lo^ (C) CD4^+^ or CD8^+^ T cells in the spleen was determined for each group of mice. (D) The capacity of CD4^+^ and CD8^+^ T cells to produce IFNγ was evaluated following simulation with PMA and ionomycin and ICCS. The percentage of CD4^+^ or CD8^+^ T cells that produce IFNγ is shown. All data are representative of 2 independent experiments, and show the mean+SE for 3 mice per group. * p<0.05, ** p<0.01, *** p<0.001, compared to the naïve (uninfected) control.

**Figure 2 ppat-1000618-g002:**
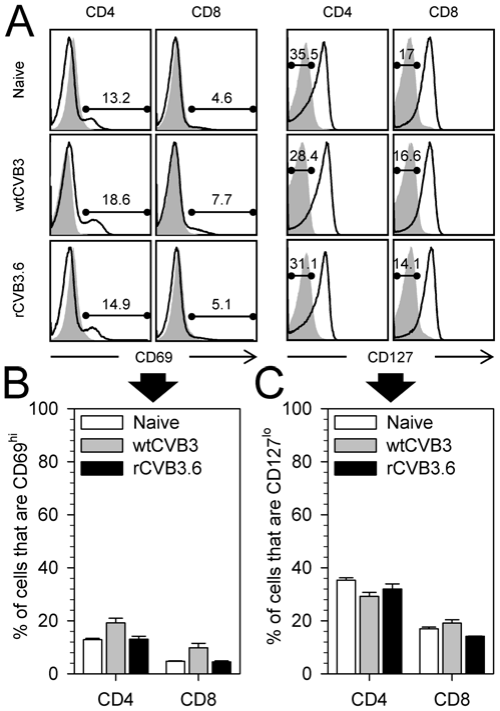
No significant change in CD69 or CD127 expression on T cells 8 days after CVB3 infection. Mice were infected with wtCVB3 or rCVB3.6, and the extent of T cell activation was analyzed on day 8. (A) CD69 and CD127 expression profiles of CD4^+^ and CD8^+^ splenocytes were compared for naïve and virus-infected mice. CD4^+^ and CD8^+^ cells were gated, and representative histograms are shown. CD69/CD127 staining is shown by solid black lines, and isotype control staining is shown in gray. The numbers indicate the percentage of gated T cells that are CD69^hi^ (left columns) or CD127^lo^ (right columns). The frequency of CD69^hi^ (B) or CD127^lo^ (C) CD4^+^ or CD8^+^ T cells in the spleen was determined for each group of mice. Data are representative of 2 independent experiments, and show the mean + SE for 3–11 mice per group; statistical analyses (ANOVA) revealed no significant differences among the groups.

The goal of this study was to evaluate CD4^+^ and CD8^+^ T cell responses to, and MHC class I and II antigen presentation by, CVB3 *in vivo*. Because native murine T cell epitopes have not been identified for CVB3, we constructed a series of recombinant CVB3 (Table 1) that encode well characterized CD8^+^ and/or CD4^+^ T cell epitopes derived from lymphocytic choriomeningitis virus (LCMV). These recombinant viruses replicate to high titers *in vivo*, but they are somewhat attenuated and are cleared moderately faster than the wildtype virus (wtCVB3) [11],[12]. Thus, before using rCVB3 to carry out analyses of epitope-specific T cell responses and antigen presentation *in vivo*, we felt it important to determine if wildtype and rCVB3 induced a similar level of overall T cell activation. Mice were infected with wtCVB3, rCVB3.6, or LCMV (as a positive control) and, 8 days later, T cell activation was broadly assessed by changes in activation marker expression and by PMA/ionomycin stimulation. Fully activated T cells are characterized by up-regulation of CD44 and down-regulation of CD62L, and these changes were readily evident in LCMV-infected mice (Figure 1A–C). In contrast, and consistent with previous work from this lab [12],[13], neither wildtype nor rCVB3 induced these coordinate phenotypic changes in CD4^+^ or CD8^+^ T cells. Some CD8^+^ T cells did show isolated up-regulation of CD44 (Figure 1A & B), perhaps indicative of sub-optimal activation but, following incubation with PMA/ionomycin, the frequencies of IFNγ-producing CD4^+^ and CD8^+^ T cells from mice infected with wtCVB3 or with rCVB3 were similar, and not significantly different from naïve control mice (Figure 1D). In contrast, a statistically-significant increase in the percentages of IFNγ^+^ CD4^+^ and CD8^+^ T cells was seen in LCMV-infected mice (Figure 1D). Furthermore, no significant changes in total numbers of IFNγ^+^ CD4^+^ or CD8^+^ T cells were observed in mice infected with wtCVB3 or with rCVB3, whereas LCMV infection led to a substantial increase in both (data not shown). We considered whether CVB3 might activate other T cell subsets (e.g. Th2 or Th17), but very few IL-4^+^ or IL-17A^+^ CD4^+^ or CD8^+^ T cells were detectable following PMA/ionomycin stimulation of splenocytes from naïve or virus-infected mice (data not shown). The lack of profound T cell activation, as judged by the foregoing criteria, was surprising, especially when one considers the extremely high titers to which CVB3 replicates *in vivo* (reaching ∼10^10^ PFU/gram in some tissues). Therefore, we carried out additional analyses, displayed in Figure 2. T cell activation often is reflected by an increase in CD69, and by a decrease in CD127 (IL-7 receptor α); when these markers were measured on CD4^+^ and CD8^+^ T cells at 8 days after CVB3 infection, no statistically-significant changes were observed. Taken together, the data in Figure 1 and Figure 2 show that both wtCVB3 and rCVB3 infections are associated with minimal activation of either CD4^+^ or CD8^+^ T cells. Our findings are consistent with a recent study which found that the proportions of splenic CD4^+^ and CD8^+^ T cells do not substantially differ between uninfected and wtCVB3-infected C57BL/6 mice [14]. In summary, rCVB3 appears similar to wtCVB3 in the extent to which infection causes activation of T cells; both viruses appear capable of largely evading detection by naïve T cells.

**Table 1:** Recombinant CVB3 encoding CD8+ or CD4+ (MHC class I/II) epitopes.

Virus name	Epitope amino acid sequence	from LCMV	T cell type	MHC allele
rCVB3.2^a^	KAVYNFATC	GP_33–41_	CD8	D^b^ (C57BL/6)
rCVB3.3^a^	RPQASGVYM	NP_118–126_	CD8	L^d^ (BALB/c)
rCVB3.4^b^	GLKGPDIYKGVYQFKSVEFD	GP_61–80_	CD4	I-A^b^ (C57BL/6)
rCVB3.5^b^	SGEGWPYIACRTSIVGRAWE	NP_309–328_	CD4	I-A^b^ (C57BL/6)
rCVB3.6^c^	KAVYNFATC GLKGPDIYKGVYQFKSVEFD	GP_33–41_ & GP_61–80_	CD8 & CD4	D^b^/I-A^b^ (see above)

a described in [11].

b described in this paper.

c described in [12].

### Longitudinal analyses show that virus-specific CD4^+^ T cells, but not virus-specific CD8^+^ T cells, expand during the course of CVB3 infection

The above experiment was somewhat limited because it used broad criteria of T cell activation to evaluate CVB3-induced T cell responses at a single time point post infection (p.i.). To assess the possibility that CVB3 infection might induce delayed T cell responses, as described in other infections [15], the kinetics of CD4^+^ and CD8^+^ T cell responses were determined by a longitudinal analysis. In order to enhance the sensitivity of detection, adoptively transferred CD8^+^ and CD4^+^ TCR-transgenic T cells were used as *in vivo* indicators of CVB3-induced T cell responses. The adoptive transfer of a large number of transgenic T cells can influence the magnitude, kinetics, survival, differentiation, and phenotype of CD8^+^ and CD4^+^ T cell responses [16]–[21]. Therefore, a small number of transgenic cells (10^4^) was used in these, and in most subsequent, experiments; we and others have shown that this number gives rise to a monoclonal T cell response that kinetically, phenotypically, and functionally resembles the endogenous, polyclonal response [18], [19], [21]–[23].

**Figure 3 ppat-1000618-g003:**
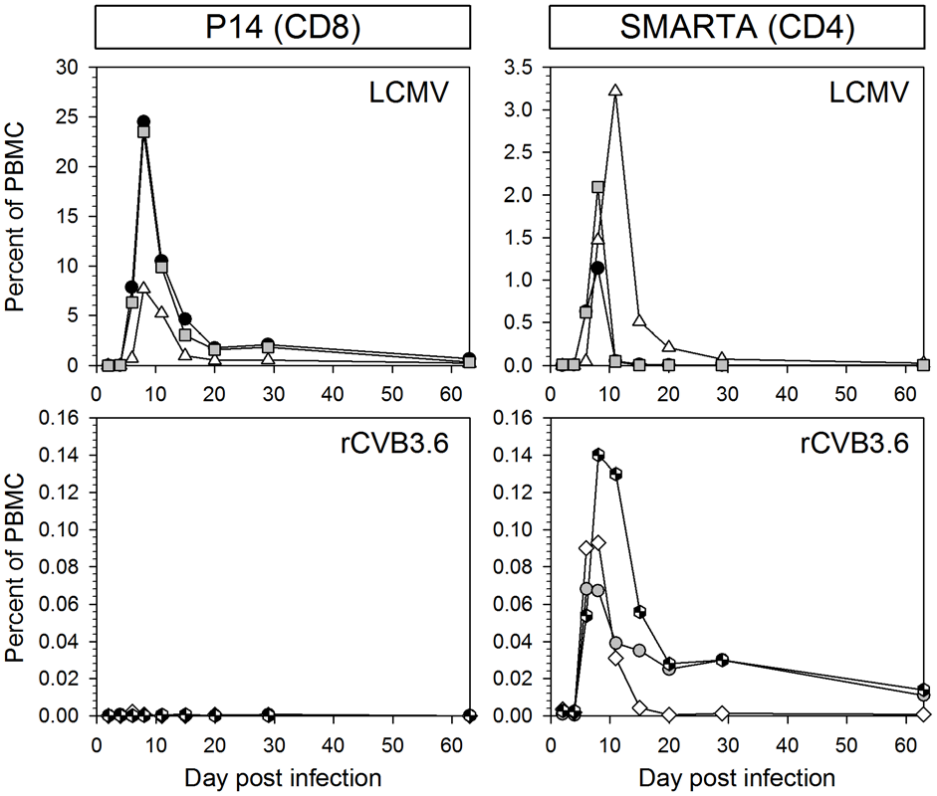
Kinetics of CD4^+^ and CD8^+^ transgenic T cell responses in rCVB3-infected and LCMV-infected mice. 10^4^ P14 cells and 10^4^ SMARTA transgenic T cells from uninfected mice were combined and transferred into uninfected congenic recipients. One day post transfer, mice were infected with rCVB3.6 or LCMV. On the indicated days p.i., the frequencies of P14 and SMARTA cells were determined by flow cytometry, and are presented as a percentage of total PBMCs. Each symbol represents the time-course in an individual mouse. Note that the y-axes differ among the graphs; this was done to facilitate the comparison of the kinetics of T cell responses that differ substantially in their magnitudes.

Mice were inoculated with naïve P14 and SMARTA cells (10^4^ of each cell type); estimating a 10% “take”, the resultant population of naïve P14 and SMARTA cells (∼10^3^ of each type) is approximately 10-fold higher than that of endogenous epitope-specific cells (estimated to be ∼100 cells/mouse [24],[25]). The following day, the mice were infected with rCVB3.6, or LCMV as a positive control, and the frequencies of transgenic cells were longitudinally monitored for 2 months. In LCMV-infected mice (Figure 3, top row), P14 cells became detectable on day 6 p.i., expanded to a peak on day 8, and then contracted to form a long-lived memory population. SMARTA cells expanded and contracted with similar kinetics, and memory cells were detectable for >60 days post LCMV infection. In striking contrast, a P14 response was not detected in CVB3-infected mice at any time point (Figure 3, bottom left). rCVB3.6 established a productive infection in these mice, however, because high fecal titers (0.41–1.27×10^6^ PFU/g) were measured on day 2 p.i. Furthermore, rCVB3.6 infection induced the expansion of SMARTA cells in these same hosts (Figure 3, bottom right), indicating that the N-terminal component of the rCVB3.6 polyprotein, containing the CD8^+^ and CD4^+^ T cell epitopes, was present in sufficient abundance to trigger expansion of naïve SMARTA cells. The CVB3-specific SMARTA response was much weaker than the response to LCMV, but showed similar kinetics, and memory SMARTA cells were detected in rCVB3.6-immune mice for >60 days p.i.. Taken together, these data suggest that there is a marked difference in the capacity of CVB3 to trigger virus-specific CD4^+^ and CD8^+^ T cell responses. In addition, we show here for the first time that virus-specific memory CD4^+^ T cells are generated after CVB3 infection.

### The absence of detectable *in vivo* CD8^+^ T cell responses cannot be attributed to redistribution into non-lymphoid organs

**Figure 4:**
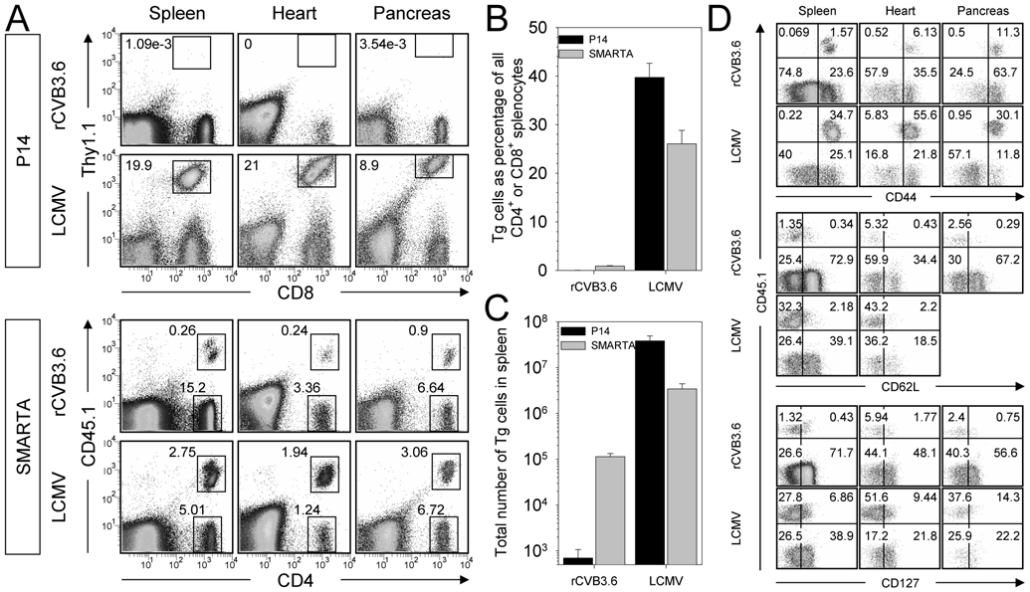
The absence of CVB3-induced CD8^+^ T cell responses cannot be attributed to redistribution into non-lymphoid target organs. Equal numbers (10^4^) of P14 and SMARTA cells from uninfected mice were combined and transferred into uninfected recipients. Four days post transfer, mice were infected with rCVB3.6 or LCMV and, 8 days later, mononuclear cells were isolated from the spleen, heart, and pancreas. (A) P14 and SMARTA responses were analyzed by flow cytometry. The gates shown in the dot plots identify P14, SMARTA, or CD4^+^ SMARTA^−^ cells, and the numbers indicate their percentage among all mononuclear cells. (B) Frequency of P14 and SMARTA cells as a percentage of relevant cells (CD8^+^ or CD4^+^) in the spleen. (C) Total number of P14 and SMARTA cells in the spleen. Data are shown as the mean+SD of 3 or 4 mice per group. (D) The expression of CD44, CD62L, and CD127 on SMARTA cells from the spleen, heart, and pancreas was compared in rCVB3.6-infected and LCMV-infected mice. The dot plots shown are gated on CD4^+^ cells, and the numbers indicate the proportion of cells in each quadrant, as a percentage of all CD4^+^ cells. CD45.1^+^ SMARTA cells are present in the upper right and left quadrants. All data are representative of 2 independent experiments.

CVB3-specific CD8^+^ T cells are barely detectable in the spleen at day 8 (Figure 1), and are undetectable in the blood at any time point p.i., even in mice that contained many P14 precursor cells (Figure 3). It remained possible that a P14 response might be induced, but could not be detected in the blood or the spleen because it was redistributed to, and constrained within, peripheral tissues such as heart and pancreas, which are major sites of CVB3 infection. However, P14 cells were barely detectable in the spleen, heart, and pancreas of rCVB3-infected mice on day 8 p.i., whereas a substantial P14 response was detected in LCMV-infected mice (Figure 4A). In contrast, a SMARTA response was observed in all tissues following infection with either rCVB3.6 or LCMV. SMARTA cells were enriched in the heart and pancreas of rCVB3.6-infected mice (∼6.7% and ∼12% of all CD4^+^ T cells, respectively) as compared to the spleen (∼1.7%), perhaps as a consequence of the high CVB3 titers that are detected in these two tissues; this selective accumulation of CVB3-specific CD4^+^ T cells further underlines the paucity of the CD8^+^ T cell response even in these virus-rich tissues. The magnitude of the CVB3-induced CD4^+^ T cell response was substantially less than the response to LCMV; both the frequency and total number of splenic SMARTA cells was ∼30-fold greater in LCMV-infected mice (Figure 4B & C). Nevertheless, the ∼10^3^ input SMARTA cells had expanded in response to rCVB3.6; there were ∼10^5^ SMARTA cells in the spleen by day 8 (Figure 4C). The great majority of rCVB3-specific SMARTA cells had an effector phenotype (CD44^hi^ CD62L^lo^ CD127^lo^) (Figure 4D), which was similar to LCMV-specific SMARTA cells, indicating that primary CD4^+^ T cells induced by rCVB3.6 infection, although low in quantity, appear to be normal in quality; additional qualitative studies are presented later in this report (see Figure eight).

### The absence of a detectable primary *in vivo* CD8^+^ T cell response to CVB3 cannot be explained by virus-mediated global suppression of CD8^+^ T cell responses

**Figure 5:**
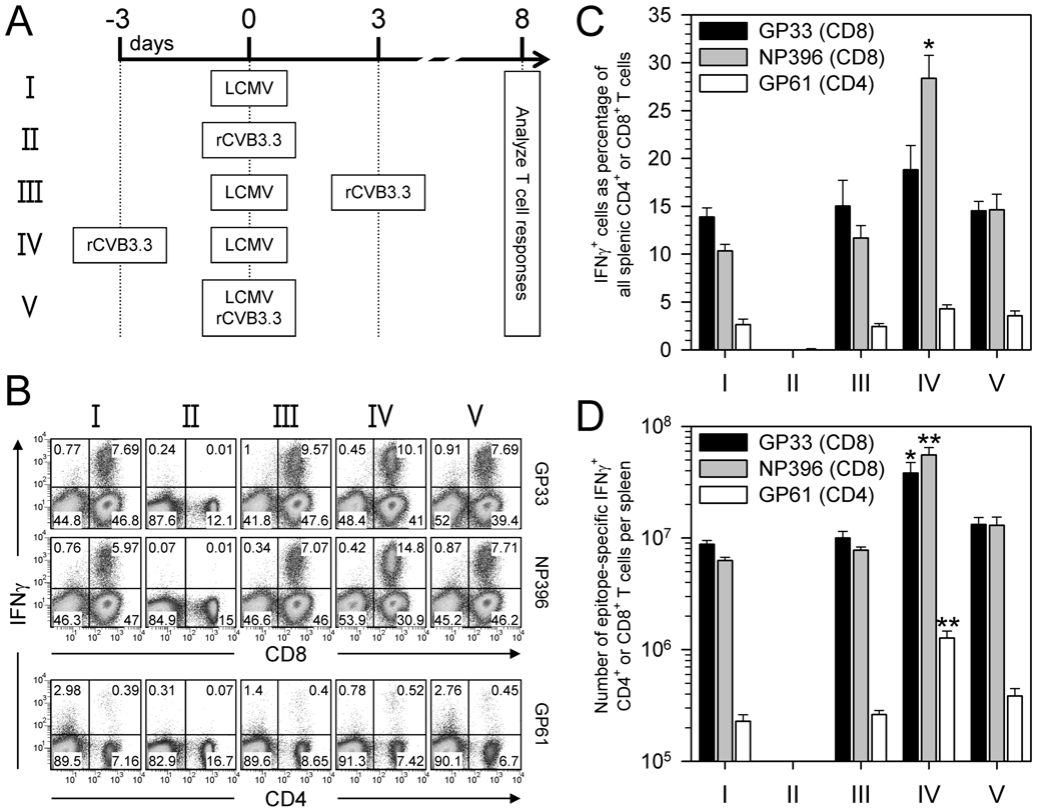
CVB3 infection does not globally suppress the host CD8^+^ T cell response. (A) Groups of mice were infected with LCMV or rCVB3.3, or co-infected with these viruses as shown. Spleens were harvested 8 days after rCVB3.3 infection (group II) or 8 days after LCMV infection (all other groups), and LCMV-specific CD8^+^ and CD4^+^ T cell responses were analyzed. (B) Splenocytes were stimulated with peptide and the frequencies of GP_33_-, NP_396_-, and GP_61_-specific IFNγ^+^ T cells were determined by flow cytometry. Representative dot plots are gated on mononuclear cells, and the numbers indicate the proportion of cells in each quadrant, as a percentage of total gated cells. (C) Percentages of epitope-specific CD8^+^ or CD4^+^ T cells that produced IFNγ; * p<0.01 compared to all other groups. (D) Total numbers of epitope-specific IFNγ-producing CD8^+^ or CD4^+^ T cells; * p<0.05, ** p<0.01 compared to all other groups. Data are shown as the mean+SE of 3 mice per group.

P14 cells might not respond to rCVB3.6 infection because the virus ablates the ability of an infected animal to mount CD8^+^ T cell responses. This could occur if, for example, CVB3 globally inactivates antigen presenting cells (APCs). To address this possibility, co-infection experiments were carried out. LCMV was selected to drive the “indicator” T cell responses, and the effects of CVB3 co-infection on these responses were measured. To ensure that the antigen-specific T cells had been triggered by LCMV antigen presentation, and not by CVB3, the co-infected mice were given rCVB3.3 [11], which does not contain relevant LCMV epitopes (see Table 1). Five groups of mice were studied, differing in co-infection status as shown in Figure 5A. Groups I and II were single-infection controls, and groups III–V were co-infections that differed in the temporal relationship between LCMV and rCVB3.3 inoculations. As expected, mice inoculated only with LCMV (group I) mounted robust CD8^+^ and CD4^+^ T cell responses (Figure 5B), while no primary responses were detected in mice infected with rCVB3.3 alone (group II). LCMV-driven T cell responses remained strong in all 3 groups of mice that were co-infected with rCVB3.3 (groups III–V, Figure 5B). Compared to mice that received only LCMV (group I), both the proportions and the absolute numbers of LCMV-specific IFNγ^+^ CD4^+^ and CD8^+^ T cells were similar in mice that had received rCVB3.3 three days after, or concurrently with, LCMV (groups III & V respectively, Figure 5C & D). Mice that received rCVB3.3 three days prior to LCMV (group IV) showed a ∼3-fold increase in the total number of splenocytes (compared to group I, data not shown), and a statistically-significant increase in epitope-specific CD4^+^ and CD8^+^ T cells (Figure 5D). These data indicate that the extraordinarily weak primary CVB3-specific CD8^+^ T cell response cannot be attributed to a global suppression of CD8^+^ T cell responsiveness; rather, the group IV data suggest that CVB3 infection may establish a microenvironment that promotes strong T cell responses, despite which the host remains incapable of mounting a response to CVB3.

### An *in vitro* antigen presentation assay shows that rCVB3.6 drives the division of CD4^+^ T cells, but not of CD8^+^ T cells

**Figure 6:**
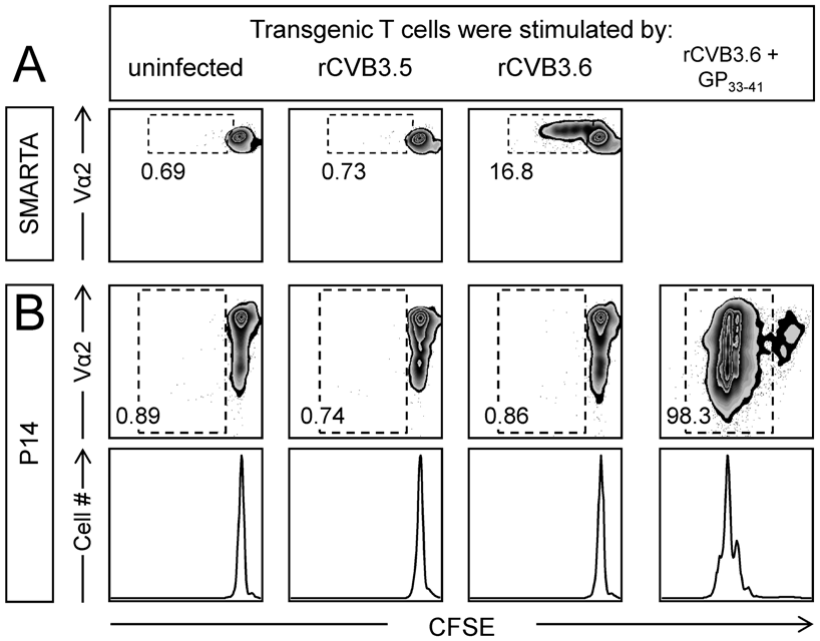
rCVB3.6 drives the *in vitro* proliferation of CD4^+^ T cells, but not of CD8^+^ T cells. Stimulator cells: Splenocytes from wt C57BL/6 mice were left uninfected, or were infected with rCVB3.5 or rCVB3.6 (moi = 10). Some splenocytes (uninfected or rCVB3.6-infected) were pulsed with GP_33–41_ peptide. Indicator cells: P14 and SMARTA splenocytes were CFSE-labeled, then mixed to generate a 1∶1 ratio of both types of transgenic T cell. Indicator cells were added to wells containing stimulator cells and, 72 hours later, wells were harvested and analyzed by flow cytometry using FloJo. The panels shown are gated on CD8^+^/Thy1.1^+^ cells (P14) or CD4^+^/CD45.1^+^/Vα2^+^ cells (SMARTA). P14 cells were not gated on Vα2 because this molecule is down-regulated on activated/dividing P14 cells (as shown in the peptide-stimulated population in panel B); had we gated on Vα2^hi^ cells, we would have failed to detect a large proportion of dividing cells. All analyses were done in triplicate, and the data shown are representative. The numbers are the proportion of (A) SMARTA cells or (B) P14 cells that have divided at least once, expressed as a percentage of total number of SMARTA or P14 transgenic T cells that were present in the plots.

One attractive explanation for the difference between CVB3-specific CD4^+^ and CD8^+^ T cell induction is that viral epitope presentation by MHC class I is limited, failing to exceed the threshold required to trigger naïve T cell responses, while antigen presentation by MHC class II remains intact, thereby stimulating virus-specific CD4^+^ T cell responses. We first assessed this hypothesis *in vitro*, using naïve CFSE-labeled P14 and SMARTA cells as sensors of CVB3 antigen presentation by infected splenocytes. Three groups of stimulator cells were prepared: (i) uninfected splenocytes; (ii) splenocytes infected at high moi with rCVB3.6; and (iii) splenocytes infected at high moi with rCVB3.5, a recombinant virus that encodes the LCMV CD4^+^ T cell epitope NP_309–328_ (see Table 1). rCVB3.5 cannot drive antigen-specific proliferation of P14 or SMARTA cells and, therefore, acts as control for bystander activation. Equal numbers of CFSE-labeled P14 and SMARTA cells were mixed and added to these three groups of stimulator cells and, three days later, the CFSE status of the P14 and SMARTA cells was assessed by flow cytometry. As shown in Figure 6A, a substantial proportion of SMARTA cells (16.8%) had divided 1–4 times in response to rCVB3.6 infection, indicating that the encoded GP_61–80_ sequence had been appropriately synthesized, processed, and presented by the infected stimulator cells. In stark contrast, the P14 cells in the same tissue culture wells showed no division (Figure 6B), consistent with the hypothesis that antigen presentation by the MHC class I pathway is compromised, preventing the stimulation of naïve CD8^+^ T cells by CVB-encoded antigen. We considered an alternative explanation: perhaps the GP_33–41_ epitope encoded by rCVB3.6 was appropriately presented to P14 cells, but these cells' subsequent division was, in some unidentified way, inhibited by rCVB3.6. To test this possibility, GP_33–41_ peptide was included in some of the rCVB3.6-infected stimulator wells (see Materials and Methods). As shown in the rightmost panels of Figure 6B, the P14 cells proliferated dramatically (the number of divisions was the same as in uninfected peptide-primed wells, not shown). Thus, rCVB3.6 infection does not suppress the division of GP_33_-specific CD8^+^ T cells, at least *in vitro*.

### rCVB3 infection drives the *in vivo* division of epitope-specific CD4^+^ T cells, but not of epitope-specific CD8^+^ T cells

**Figure 7:**
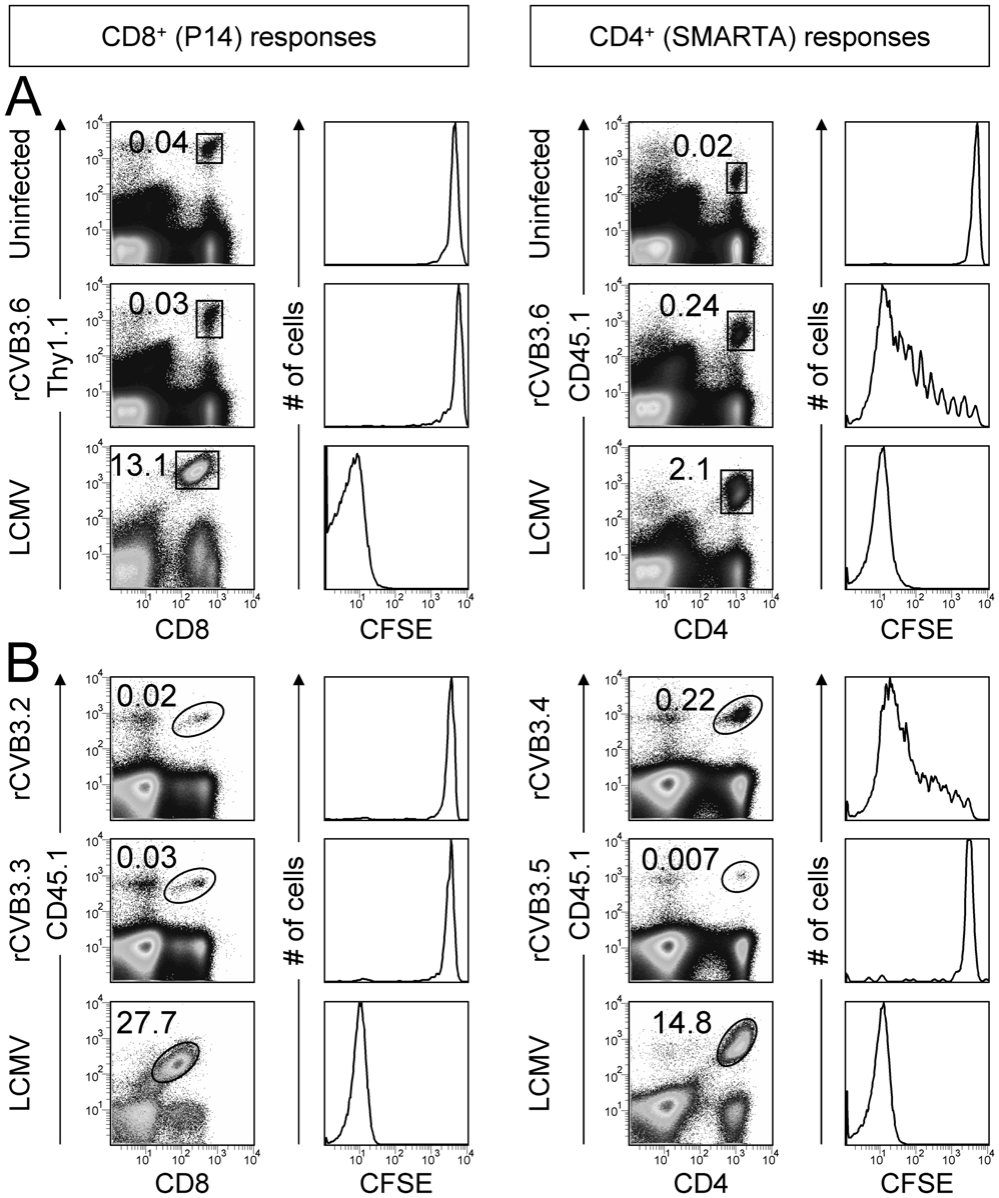
CVB3 infection induces the *in vivo* proliferation of virus-specific CD4^+^, but not CD8^+^, transgenic T cells. (A) Equal numbers (6×10^5^) of P14 and SMARTA cells from uninfected mice were labeled with CFSE, combined, and transferred into uninfected recipients. One day post transfer, mice were infected with rCVB3.6 or LCMV, or were not infected. Eight days later, P14 and SMARTA responses in the spleen were analyzed by flow cytometry. The square gates in the dot plots identify P14 or SMARTA cells, and the numbers shown are the percentages of transgenic cells among mononuclear cells. The histograms of CFSE fluorescence are gated on P14 or SMARTA cells. Data are representative of 3 mice per group. (B) Mice were inoculated with rCVB3.2, 3.3, 3.4, or 3.5. Two days after infection, naïve P14 and SMARTA cells were labeled with CFSE, and 9×10^5^ transgenic T cells (P14 or SMARTA) were injected i.v. into the infected mice. Eight days post transfer (10 days p.i.) the P14 or SMARTA cells were identified by flow cytometry (ovals within dot plots). The histograms show the level of CFSE fluorescence by the donor cells. As a positive control, the CFSE-labeled transgenic T cells were given to uninfected mice; the following day these mice were infected with LCMV, and 7 days later these cells were identified (bottom row, dot plots) and their near-total loss of CFSE-fluorescence was revealed (bottom row, histograms).

*In vitro* assays, although persuasive, are intrinsically artificial, and we considered it important to use the transgenic T cells as sensors of antigen presentation *in vivo*. To this end, CFSE-labeled P14 and SMARTA cells were adoptively transferred into mice; the proliferation of these transgenic cells provides a functional readout of the *in vivo* presentation of CVB-encoded epitopes by MHC class I and II. Individual mice that had received both P14 and SMARTA T cells were inoculated with rCVB3.6, or with LCMV as a positive control; rCVB3.6-inoculated mice had a high fecal titer on day 2, which confirmed that the mice had been successfully infected. A low frequency of P14 and SMARTA cells was detected in uninfected mice and little if any proliferation of P14 and SMARTA cells occurred (Figure 7A, top row). In contrast, P14 and SMARTA cells expanded to a much higher frequency in LCMV-infected mice by day 8, and nearly all these cells had divided >7 times (Figure 7A, bottom row); this result confirms that the input cells serve as indicators of antigen presentation. In stark contrast, P14 cells did not proliferate in rCVB3.6-infected animals (Figure 7A, middle row); almost all of the P14 indicator cells remained CFSE^hi^. Furthermore, the numbers of CFSE-labeled P14 cells recovered from naïve mice and from rCVB3.6-infected mice were almost identical (p = 0.27), indicating that rCVB3.6 had not triggered apoptosis of the input sensor cells (data not shown). Thus, during rCVB3.6 infection, presentation of the encoded GP_33–41_ epitope is insufficient to trigger the division of P14 cells. In contrast, SMARTA cells showed some proliferation (Figure 7A, middle row), consistent with our proposal above.

It was important to confirm that the distinction between CD4^+^ and CD8^+^ T cell division was not limited to one recombinant virus, rCVB3.6; and also to demonstrate that the CVB3-induced SMARTA response was epitope-specific, rather than a bystander effect. Therefore a similar approach was taken using four additional rCVB3 (Table 1). One pair of viruses (rCVB3.2 & 3.3) encode single CD8^+^ T cell epitopes, while the second pair (rCVB3.4 & 3.5) encode single CD4^+^ T cell epitopes. Within each pair, one virus encodes an epitope that could trigger the sensor T cells, while the other does not (thereby acting as a control for epitope specificity/bystander stimulation). To evaluate epitope presentation by MHC class I, mice were inoculated with rCVB3.2 or 3.3, and then received naive CFSE-labeled P14 cells 2 days later. Eight days post transfer (10 days p.i.), the frequency of P14 cells was almost identical in mice infected with rCVB3.2 (which encodes GP_33–41_) or rCVB3.3 (which does not encode that epitope), and these cells underwent essentially no cell division and remained CFSE^hi^ (Figure 7B, left columns); thus, rCVB3.2 did not drive the epitope-specific expansion of P14 sensors. Parallel experiments assessed epitope presentation by MHC class II, using rCVB3.4, rCVB3.5 and CFSE-labeled SMARTA cells (Figure 7B, right columns). SMARTA cells underwent several cycles of division in mice that were infected with rCVB3.4 (which encodes GP_61–80_) and this response was epitope-specific, because it was absent from mice infected with the negative control virus (rCVB3.5). A positive control, using LCMV, confirmed the responsiveness of SMARTA cells (bottom row). This striking contrast in proliferation of CVB3-specific CD8^+^ versus CD4^+^ T cells mirrors the data in Figure 7A. Taken together, the *in vitro* (Figure 6) and *in vivo* (Figure 7) data strongly suggest that epitope presentation by the MHC class I pathway is almost entirely ablated in CVB3-infected cells, while epitope presentation by the MHC class II pathway remains partially intact.

### SMARTA T cell responses to rCVB3.6 are an accurate reflection of endogenous CD4^+^ T cell responses

**Figure 8:**
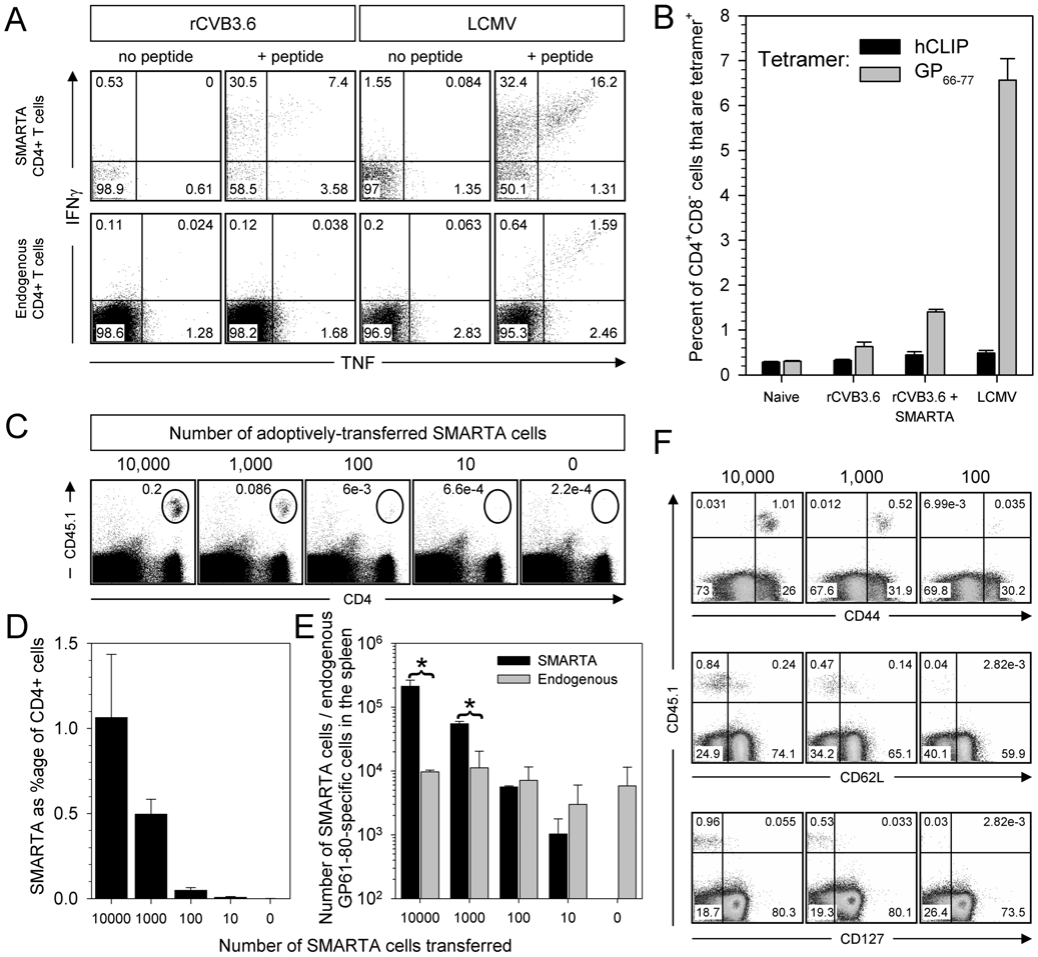
SMARTA T cell responses to rCVB3.6 provide an accurate reflection of the responses of endogenous epitope-specific CD4^+^ T cells. (A) The capacity of transgenic and endogenous CD4^+^ T cells to produce IFNγ and TNF was evaluated. Dot plots are gated on SMARTA cells (top row) or on endogenous CD4^+^ T cells (bottom row); the numbers indicate the proportion of cytokine-producing cells in each quadrant, as a percentage of total gated cells. (B) The frequency of splenic GP_61_-specific CD4^+^ T cells was enumerated using I-A^b^/GP_66–77_ MHC class II tetramers. I-A^b^/hCLIP tetramers were included as a negative control. (C) Differing numbers of naïve SMARTA cells [10^4^; 10^3^; 10^2^; 10^1^; or none (media only)] were transferred into uninfected mice, and 4 days after transfer, the mice were infected with rCVB3.6. Eight days p.i., the SMARTA response in the spleen was analyzed by flow cytometry. The oval gates in the dot plots identify SMARTA cells, and the numbers shown are the percentage of SMARTA cells among all mononuclear cells. (D) Frequency of SMARTA cells as a percentage of CD4^+^ splenocytes. (E) Total numbers of SMARTA cells (measured by surface staining for CD4 and CD45.1) and GP_61–80_-specific endogenous cells (measured by IFNγ ICCS) in the spleen. Data are shown as the mean+SD of 3 mice per group, * p<0.005. (F) The expression of CD44, CD62L, and CD127 on SMARTA cells was compared among rCVB3.6-infected mice that had received 10^4^, 10^3^, or 10^2^ transgenic cells. Dot plots are gated on CD4^+^ cells, and the numbers indicate the proportion of cells in each quadrant, as a percentage of CD4^+^ cells. Data are representative of two independent experiments.

The above data (Figure 3, 4, 6, 7) using P14 and SMARTA cells revealed a profound difference between CD8^+^ and CD4^+^ T cell responses to rCVB3 infection. To ensure that the more pronounced responses by SMARTA cells were not an artifact of their being TCR-transgenic, and/or a function of their artificially-high precursor frequency in recipient mice, we next compared the responsiveness of SMARTA cells to their endogenous counterparts. Since minimal endogenous CD4^+^ T cell responses to CVB3 were observed using broad measures of T cell activation (Figure 1), we used more sensitive approaches to evaluate the endogenous GP_61–80_ response, including ICCS and MHC class II tetramers. Mice received a mixture of P14 and SMARTA cells (10^4^ of each) and were then infected with rCVB3.6 or LCMV. In LCMV-infected mice (day 8 p.i.), a substantial percentage of SMARTA cells (∼49.9%) and endogenous CD4^+^ T cells (∼4.7%) produced IFNγ and/or TNF in response to GP_61–80_ peptide stimulation (Figure 8A). A substantial proportion of SMARTA cells from CVB3-infected mice also produced IFNγ and/or TNF (∼41.5%), and a proportion of SMARTA cells also produced IL-2 in rCVB3-infected (∼25%) and LCMV-infected (∼35%) mice (data not shown). The endogenous GP_61–80_-specific CD4^+^ T cell response was difficult to detect in these mice by ICCS (Figure 8A, lower left dotplots) and we considered the possibility that rCVB3.6 might induce dysfunctional CD4^+^ T cells. Therefore, we used I-A^b^/GP_66–77_ MHC class II tetramers to enumerate GP_61–80_-specific CD4^+^ T cells regardless of their function; background binding was determined using a control tetramer, I-A^b^/hCLIP. As expected (Figure 8B), a large population of I-A^b^/GP_66–77_ tetramer^+^CD4^+^CD8^−^ T cells was identified in LCMV-infected mice (without adoptively-transferred cells). In contrast, CVB3-infected mice contained a very small population of I-A^b^/GP_66–77_ tetramer^+^CD4^+^ T cells (∼0.3% above background). An additional group of animals that received 10^4^ SMARTA cells prior to infection with rCVB3.6 showed, as expected, a higher percentage of I-A^b^/GP_66–77_ tetramer^+^CD4^+^CD8^−^ T cells (∼1% above background) compared to rCVB3.6-infected mice that had not received SMARTA cells (Figure 8B); these tetramer^+^ cells were mostly CD45.1^+^ (SMARTA) cells (data not shown). Overall, MHC class II tetramer staining did not reveal a large number of non-functional (cytokine negative) endogenous GP_61–80_-specific T cells in CVB3-infected mice. Rather, there is a good correlation between the responses detected by tetramer and ICCS assays.

The very low frequency of rCVB3.6-induced endogenous CD4^+^ T cells prevented a direct comparison of the antigen-responsiveness of these cells to SMARTA cells. We hypothesized that, if naïve SMARTA and endogenous cells were similarly responsive to antigen, then by reducing the precursor frequency of SMARTA cells to endogenous CD4^+^ T cell precursor frequency, the ensuing SMARTA response to CVB3 should become almost undetectable (i.e., similar to the endogenous response). Mice received differing numbers of naïve SMARTA cells (10^4^; 10^3^; 10^2^; 10^1^; or none), and were then infected with rCVB3.6. All mice in all 5 groups had a high virus titer in the feces (∼10^6^–10^7^ PFU/g) on day 2 p.i. As expected, a sizable SMARTA response was detected on day 8 in mice that had received 10^4^ transgenic cells, and the response declined as the number of input cells decreased (Figure 8C). The frequency of SMARTA cells was dramatically diminished in mice that had received ≤100 SMARTA cells (Figure 8D), and reached the limit of detection in mice that had received 10 transgenic cells. The total number of GP_61–80_-specific endogenous CD4^+^ T cells in the spleen was quite similar in all groups regardless of the number of SMARTA cells transferred (Figure 8E). The total number of SMARTA cells was significantly (p<0.005) greater than the number of endogenous GP_61_-specific CD4^+^ T cells in mice that had received 10^4^ or 10^3^ transgenic cells, but no statistically-significant difference in the magnitude of SMARTA and endogenous responses was present in rCVB3.6-infected mice that had received 100 SMARTA cells. This laboratory has previously shown, using LCMV, that C57BL/6 mice contain ∼100 GP_61–80_-specific precursor CD4^+^ T cells [25]. Therefore, we conclude that, when SMARTA cells and endogenous cells are at approximately the same precursor frequency, they mount responses of similar magnitude; thus, SMARTA cells provide an accurate reflection of endogenous CD4^+^ T cell proliferation. Next, we asked if SMARTA cells also provided an accurate estimate of T cell quality. T cell phenotype can be partially dependent upon the frequency of naïve precursors [19],[21], and we thought it relevant to determine if the phenotype of rCVB3-induced SMARTA cells (Figure 4D) was greatly altered by the naïve precursor frequency. The majority of SMARTA cells were CD44^hi^CD62L^lo^CD127^lo^ at day 8, and the proportions were very similar among mice with differing naïve SMARTA precursor frequencies (Figure 8F). Furthermore, the proportions of SMARTA cells that produced IFN-γ, IL-2, or TNF on day 8 were similar when 10^3^ or 10^4^ SMARTA cells were transferred before CVB3 infection, and neither SMARTA cells nor endogenous GP_61–80_-specific CD4^+^ T cells produced IL-4 or IL-17A (data not shown). Together, these data indicate that our ability to detect a strong rCVB3-specific SMARTA cell response is not due to an intrinsic difference in the ability of endogenous GP_61–80_-specific and SMARTA cells to respond to rCVB3.6; rather, when 10^3^–10^4^ SMARTA cells are transferred, the resulting elevation in precursor frequency renders any responses more easily detected.

### rCVB3.6 infection generates functional CD4^+^ memory T cells, but not CD8^+^ memory cells

Finally, we investigated the quality and quantity of rCVB3-induced memory T cells. This was done for two reasons. *First*, as an additional means by which to evaluate the priming of P14 cells during the primary rCVB3.6 infection. Although we did not detect significant primary rCVB3-specific P14 responses, it remained possible that some P14 cells had been successfully activated, in numbers too low to allow detection, and the progeny of those cells might become memory cells that could expand to detectable levels following strong antigen stimulation (in this case, potential memory cells were stimulated by infecting the mice with LCMV). *Second*, given the apparent deficiencies in antigen presentation during CVB3 infection, it was important to determine if any resulting memory T cells were functionally normal. However, it was possible that naïve (i.e., antigen inexperienced) P14 and SMARTA cells might persist in rCVB3.6-infected mice after the virus was cleared. Thus, when challenging rCVB3.6-immune mice with LCMV, it was important to determine whether any responses were due to the activation of rCVB3.6-induced memory cells (true recall responses), or to primary responses mounted by residual naïve transgenic T cells. We addressed this issue in two ways. *First*, we included a negative control group of mice that had been infected with rCVB3.3. This virus cannot cause epitope-specific stimulation of P14 or SMARTA cells, and so any responses to LCMV challenge must represent primary responses of residual naïve transgenic cells. *Second*, we evaluated the LCMV-stimulated responses at days 4 and 7 after secondary infection, reasoning that a response detectable at day 4 most probably has been mounted by memory cells that are present at increased frequency, while a response at day 7 could be attributed either to naïve cells or memory cells (or both).

**Figure 9:**
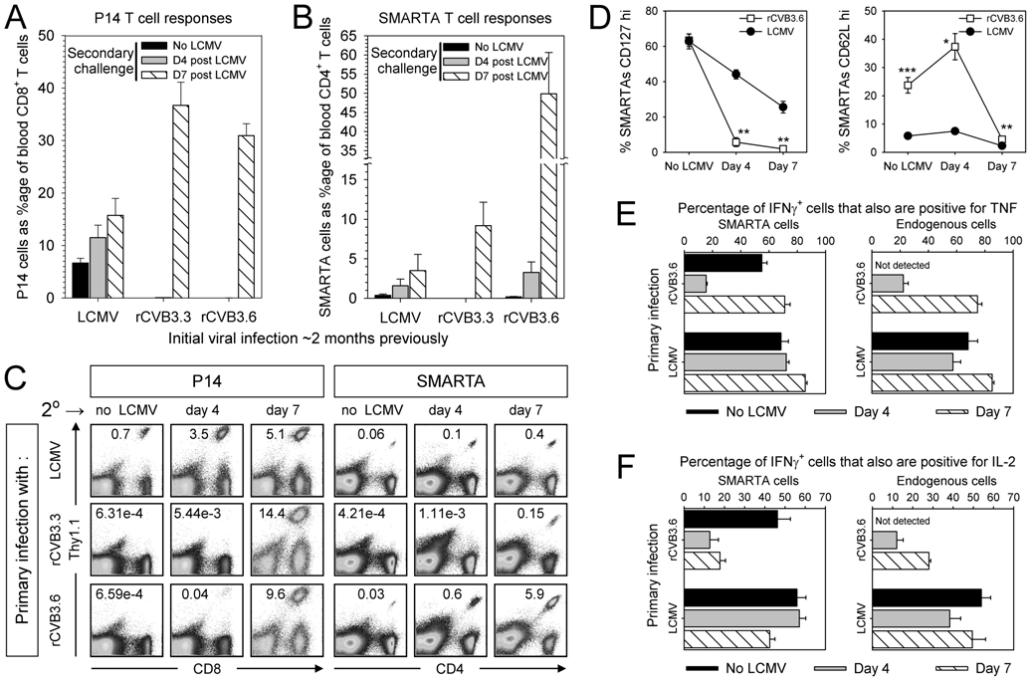
rCVB3.6 induces memory CD4^+^ T cells that expand in number and differentiate into secondary effectors following a challenge infection. Equal numbers (1×10^4^) of P14 and SMARTA cells from uninfected mice were combined and transferred into uninfected recipients. One to five days later, these mice were infected with rCVB3.3, rCVB3.6, or LCMV. Sixty to sixty-five days after the primary infection, the mice were challenged with LCMV (or left unchallenged), and P14 and SMARTA responses were analyzed on day 4 and 7 post challenge. Frequency of (A) P14 and (B) SMARTA cells as a percentage of CD8^+^ or CD4^+^ cells, respectively, in the blood of unchallenged and LCMV-challenged mice. Data are shown as the mean+SE of 6–16 mice per group, pooled from 4 independent experiments. (C) P14 and SMARTA responses in the spleens of these mice; the numbers in the dotplots indicate the percentage of transgenic cells among all mononuclear cells. (D) Changes in CD127 and CD62L expression on SMARTA cells following LCMV challenge of mice previously infected with rCVB3.6 (squares) or LCMV (circles). The percentage of SMARTA cells that were CD127^hi^ or CD62L^hi^ are shown as the mean ± SE of 2–4 mice per time point, pooled from 2 independent experiments; * p<0.05, ** p<0.005, *** p<0.001. The capacity of the transgenic and endogenous GP_61_-specific CD4^+^ T cells to produce IFNγ and TNF (E), or IFNγ and IL-2 (F) was evaluated. Data are shown as the mean + SE of 2–4 mice per group, combined from 2 independent experiments.

Mice received equal numbers of naïve P14 and SMARTA cells, then received a primary infection: LCMV (positive control group); rCVB3.3 (negative control group); or rCVB3.6. Two months later the mice were challenged with LCMV (or were left unchallenged) and T cell responses were analyzed on days 4 and 7. As expected, LCMV-immune mice contained abundant P14 cells in the blood prior to secondary challenge (Figure 9A, black bar), and these cells increased in frequency at days 4 and 7 (grey and hatched bars). In contrast, prior to LCMV infection no P14 cells were detected in the blood of mice that had been infected with rCVB3.3 or 3.6, and P14 cells remained almost undetectable in the blood at 4 days after LCMV infection of the rCVB3-immune mice, suggesting that rCVB3.6 had not induced memory cells in sufficient quantity to provide a detectable accelerated response to LCMV challenge. By 7 days after LCMV infection, strong P14 responses were present in all mice (hatched bars), but the magnitude of the response in rCVB3.3-immune mice was very similar to that observed in rCVB3.6-immune mice, suggesting that the majority of responding cells in rCVB3.6-immune animals were primary effector cells, generated from residual naïve P14 cells. Prior to LCMV challenge, low numbers of SMARTA cells were detected in LCMV-immune and rCVB3.6-immune mice, but not in rCVB3.3-immune animals, suggesting that rCVB3.6 had induced memory SMARTA cells (Figure 9B). This conclusion was supported by analyses at 4 days post-LCMV; SMARTA cell numbers increased 40-fold in rCVB3.6-immune mice, but remained undetectable in rCVB3.3-immune mice. At day 7, SMARTA cells further increased in rCVB3.6 mice, and constituted ∼50% of all CD4^+^ T cells in the blood. However, a strong SMARTA response also was present in the rCVB3.3 group, which must represent a primary response mounted by naïve cells. Consequently, we conclude that the day 7 SMARTA response in rCVB3.6 mice probably comprises a mixture of primary responders and a true recall response. Analyses of P14 and SMARTA cells in the spleen (Figure 9C, left side) largely confirmed the above conclusions. P14 cells were undetectable in rCVB3-immune mice before LCMV infection, and even at 4 days post-LCMV, very few P14 cells were present; there was no statistically-significant difference between the numbers of P14 cells that were present in rCVB3.3-immune and rCVB3.6-immune animals (p>0.16). In addition, the presence of SMARTA cells prior to LCMV infection in rCVB3.6 mice, but not in rCVB3.3 mice, was clearly demonstrable, as was their more rapid expansion after LCMV infection (Figure 9C, right side). Taken together, the data in Figure 9A–C suggest that rCVB3.6 induces memory CD4^+^ T cells that undergo expansion upon secondary encounter with their cognate antigen; but the virus does not induce a substantial number of memory CD8^+^ T cells.

Finally, we characterized rCVB3.6-specific memory CD4^+^ T cells, evaluating several of the phenotypic and functional characteristics of memory T cells, and their changes as the cells differentiate into secondary effectors [26],[27]. A similar proportion of SMARTA memory cells induced by LCMV or by rCVB3.6 were CD127^hi^ (∼63%), and both populations became predominantly CD127^lo^ in response to LCMV challenge, although the rCVB3 memory cells showed a more rapid conversion (Figure 9D, left panel). Prior to LCMV challenge, a significantly greater percentage of CVB3-specific SMARTA memory cells were CD62L^hi^ compared to LCMV-specific SMARTA cells (Figure 9D, right panel); we speculate that a lower level of MHC class II antigen presentation, and/or a weaker (or more brief) period of stimulation during rCVB3 infection, permitted more rapid CD62L expression by CVB3-induced memory CD4^+^ T cells. Following LCMV challenge, the progeny of these cells showed much-reduced expression of CD62L, and by day 7 nearly all of these cells had become CD62L^lo^. Thus, the majority of CVB3-induced memory CD4^+^ T cells become CD62L^lo^CD127^lo^ after LCMV challenge. In addition, cytokine expression profiles were used to distinguish CVB3-induced memory and secondary effector T cells, and to assess their functional capabilities. Previous studies have shown that the cytokine profile of virus-specific memory CD8^+^ T cells becomes more effector-like as these cells differentiate into secondary effector cells following re-exposure to cognate antigen [26]. Without LCMV infection, a large fraction (∼55%, Figure 9E) of the IFNγ^+^ memory SMARTA cells in rCVB3.6-immune mice co-expressed TNF in response to peptide stimulation, and this fraction was dramatically lower at 4 days after LCMV challenge, consistent with the cells' developing a secondary effector phenotype. The proportions of resting memory SMARTAs that co-produced IFNγ and IL-2 were similar whether the cells had been induced by rCVB3.6 or by LCMV (46% and 56% respectively, Figure 9F, left panel, black bars). After LCMV challenge, most of the rCVB3-induced memory SMARTA cells lost the capacity to produce IL-2, whereas the majority of LCMV-induced SMARTA cells retained the capacity to co-produce IL-2. Prior to LCMV challenge, the frequency of rCVB3-induced endogenous GP_61_-specific memory CD4^+^ T cells was too low to perform a similar analysis, but at days 4 and 7 the proportion of GP_61_-responsive endogenous cells that was double-positive was similar to that observed for the SMARTA population (Figure 9E and F). Taken together, these data show that high quality, multi-cytokine producing virus-specific memory CD4^+^ T cells are generated following CVB3 infection, which differentiate into functional secondary effector T cells upon challenge infection.

## Discussion

Our understanding of the adaptive immune response to CVB remains extraordinarily limited [28]. Early studies detected cytotoxic activity in CVB3-infected mice [29]–[31], and showed that T cells could proliferate *in vitro* when stimulated with CVB3 VP1 sequences [32], but the lack of known T cell epitopes in CVB3, and the weakness of virus-specific T cell responses has made it difficult to analyze their specificity and kinetics [3]. We have previously reported [12] that rCVB3.6 fails to induce strong endogenous primary T cell responses, and the relative weakness of CVB3-specific CD8^+^ T cell responses also has been demonstrated in human studies, in which the *ex vivo* frequency of CVB3-specific CD8^+^ T cells was so low that their detection required ∼2 weeks of *in vitro* antigen stimulation [33]. In this manuscript we have attempted to identify the reason for these weak responses, and have used transgenic T cells to assess antigen presentation by CVB3 *in vivo*. In addition, our approach has allowed us to separately evaluate the virus' effects on the MHC class I and class II antigen presentation pathways.

We report herein that CVB3, despite replicating to remarkably high titers *in vivo* (up to 10^10^ PFU/gram in some tissues), does not induce marked activation of CD4^+^ or CD8^+^ T cells. The absence of CD8^+^ T cell responses is especially dramatic: rCVB3.6 fails to induce a detectable primary CD8^+^ T cell response even when the frequency of epitope-specific precursors is artificially increased by ∼10-fold (Figure 1–[Fig ppat-1000618-g002] [Fig ppat-1000618-g003] Figure 4). Furthermore, CFSE-labeled P14 cells fail to divide in response to rCVB3.6-infected splenocytes (Figure 6) and are similarly unresponsive when transferred to mice infected with this recombinant virus, or with rCVB3.2, which also encodes the GP_33_ epitope (Figure 7). The absence of a detectable rCVB3-specific primary CD8^+^ T cell response at 8 days post-infection was not due to a delayed CD8^+^ T cell expansion (Figure 3), nor was it due to redistribution into virus-infected peripheral tissues (Figure 4). We considered the possibility that CVB3 might actively suppress the T cell response by, for example, globally disrupting antigen-presenting cells. CVB3 replication is restricted in DCs *in vitro* and *in vivo* and infectious virus is not produced [14], but a non-productive infection might still impair DC function. Moreover, several picornaviruses may limit the ability of dendritic cells (DCs) to prime robust virus-specific T cell responses, by disrupting protein trafficking [34]–[38] and thereby inhibiting expression of cytokine receptors [39],[40], costimulatory molecules [41], and MHC class I [38],[42]. However, three separate observations herein show that CVB3 causes neither a global inhibition of DC function, nor a suppression of T cell responsiveness. *First*, infected mice mount primary CD4^+^ T cell responses to rCVB3.6 (Figure 3, Figure 4), suggesting that DCs can present antigen together with the required costimulatory signals. *Second*, CVB3 infection does not diminish the CD8^+^ T cell response induced by co-infection with LCMV, and may even have increased it (Figure 5). *Third*, GP_33–41_ peptide drove the division of P14 cells in wells infected with rCVB3.6, excluding an inhibitory effect of the virus on T cell proliferation (Figure 6). Regulatory T cells might suppress strong virus-specific CD8^+^ T cell responses, but this appears unlikely because there is no significant change in CD4^+^CD25^+^FoxP3^+^ T cell frequency following infection [14],[43], and primary LCMV-specific CD8^+^ T cells response were unabated in CVB3-infected animals (Figure 5).

We conclude that the most likely explanation for the absence of a detectable primary CD8^+^ T cell response to rCVB3.6 is a block in presentation of the encoded CD8^+^ epitope *via* the MHC class I pathway. This is demonstrated by the failure of CFSE-labeled P14 cells to divide over a 3-day period in tissue culture (Figure 6) and, perhaps most dramatically, by their unresponsiveness after being incubated for 8 days in an rCVB3.6-infected mouse (Figure 7). For two reasons we can exclude the obvious concern that the GP_33–41_ component of the dual-epitope sequence is in some way flawed, and is intrinsically incapable of being processed and presented. *First*, the translation initiation codon and the GP_33–41_ epitope lie upstream of the GP_61–80_ sequence and, therefore, SMARTA cell responses to rCVB3.6 constitute proof that the CD8^+^ epitope sequence must be synthesized. *Second*, when subcloned from rCVB3.6 into a plasmid DNA vaccine, the dual-epitope sequence induced a strong epitope-specific CD8^+^ T cell response [12]. Therefore, this CD8^+^ T cell epitope sequence, when expressed independently of CVB3 proteins, can be processed and presented at a level that is sufficient to trigger responses by naïve CD8^+^ T cells, but this process is abrogated in the rCVB3.6-infected cell. A molecular explanation is available. We, and others, have shown that the CVB3 proteins 2B, 2C, and 3A cooperate to interfere with protein trafficking within an infected cell [34]–[38], and that their actions lead to a dramatic and rapid down-regulation of surface MHC class I *in vitro* [38],[42]. Consequently, we propose that these trafficking defects are responsible for the extraordinarily weak primary CD8^+^ T cell response to CVB3 infection. We cannot exclude the possibility that CVB3 exerts additional inhibitory effects on the MHC class I pathway; for example, the virus may alter antigen processing, or other steps in the pathway. It is important to note, however, that this trafficking blockade, although profound, is incomplete, because we have shown that GP_33_-specific CD8^+^ memory T cells proliferate *in vivo* in response to rCVB3.6 infection [12]. What might explain this difference between naïve and memory CD8^+^ T cells? Memory cells are better able to respond to low levels of antigen [44], and we have suggested that presentation of the rCVB3.6-encoded epitopes may lie below a threshold level that is required to trigger naïve T cells, but above the threshold required by memory cells [12]. Other explanations are possible. For example, naïve T cell precursors are triggered only by antigen presented by professional APCs, while memory T cells also can recognize antigen presented on a variety on “non-professional” cell types. It is possible that CVB3 inhibits MHC class I presentation to a greater extent in APCs than in non-professional cells, allowing the latter population to selectively stimulate responses by memory T cells. However this explanation appears unlikely, because the rapid and profound interruption in protein trafficking that has been previously reported occurs in CVB3-infected fibroblasts [34],[37],[38],[45].

Elegant analyses from several laboratories have provided strong evidence to support the concept that primary CD8^+^ T cell responses to virus infection can be induced *via* an alternate pathway of MHC class I antigen presentation. In this process, termed cross presentation/cross priming, exogenous antigen is taken up by specialized APCs, and is introduced into the MHC class I pathway and presented to naïve T cells. Cross presentation/cross priming appears to be an important contributor to the induction of antiviral CD8^+^ T cell responses to several viruses, including poxviruses and herpesviruses [46]–[49], but three findings in this report suggest that this alternate pathway does not operate efficiently during CVB infection. First, the absence of detectable primary CD8^+^ T cell responses to CVB3 (Figures 3 & 4); second, the failure of P14 cells to divide in rCVB3.6-infected tissue culture wells in which CVB-encoded antigen is being presented, as shown by the division of SMARTA cells (Figure 6); and, third, the failure of P14 cells to divide *in vivo* in rCVB3.6-infected mice (Figure 7). If cross-priming were efficient in CVB3-infected animals *in vivo*, one would expect that a potential epitope in, for example, the viral VP4 protein might be taken up from the extracellular milieu and cross-presented by MHC class I, inducing detectable CD8^+^ T cell responses (i.e., cross priming). It is unlikely that the failure of cross priming can be attributed to a lack of available extracellular antigen, because our data show that rCVB3.6-encoded antigen was sufficiently abundant to induce CD4^+^ T cell responses. It is possible that the recipient APCs also ingested the viral 2B, 2BC and 3A proteins in the appropriate stoichiometry, thereby preventing successful cross presentation. However, this is improbable, for at least two reasons. First, it is difficult to imagine that all APCs would be so affected. Second, if all APCs did take up these inhibitory proteins in a form that led to trafficking defects, one would expect that trafficking of MHC class II also would be affected within those cells; our identification of CD4^+^ T cell responses argues against this possibility. Therefore, we speculate that, for unknown reasons, cross presentation is ineffective in CVB3-infected mice. At present, we cannot provide a molecular explanation for the apparent failure of cross-priming in CVB3-infected mice; these studies are ongoing.

In contrast to CD8^+^ T cells, naïve SMARTA CD4^+^ T cells divide extensively, indicating that MHC class II-restricted viral epitopes are presented at a level sufficient to trigger CVB3-specific CD4^+^ T cells. Why, though, are CD4^+^ T cell responses not stronger? Epitopes presented by MHC class II are most often derived from uptake of exogenous proteins, but recent work has shown that class II-restricted epitopes may be derived from endogenous proteins [50]–[53]. We speculate that the “classical” exogenous route of class II presentation remains intact, while the endogenous MHC class II pathways – like the MHC class I pathway – are interrupted by CVB3 infection, thereby diminishing the capacity of encoded epitopes to induce CD4^+^ T cell responses. Nevertheless, CD4^+^ T cell responses appear to play a role in regulating the outcome of CVB infection, and recent intriguing work has suggested that CVB3-induced CD4^+^ T cells can confer some degree of protection following their transfer into a naïve host [54]. The data presented herein are, to our knowledge, the first detailed analysis of epitope-specific CD4^+^ T responses to CVB3. Previous studies in mice have reported CD4^+^ T cell responses to coxsackievirus B4, but the epitope-specific T cells were analyzed only after several days of *in vitro* restimulation [55],[56]. We show here that CVB3-specific CD4^+^ T cells display an effector phenotype and a T_h_1 cytokine profile, and are enriched among CD4^+^ T cells in peripheral sites of virus infection that are major targets of CVB3 pathogenesis. CVB3 infection drives the differentiation and generation of virus-specific memory CD4^+^ T cells; upon encountering another virus encoding their cognate antigen, these cells expand in number (>400-fold), differentiate into secondary effectors, and produce multiple antiviral and pro-survival cytokines. The differentiation and survival of memory CD4^+^ T cells may depend on the strength of antigen stimulation received in the primary response [23]. If competition for antigen stimulation is increased by elevating the precursor frequency of naïve CD4^+^ transgenic T cells or by limiting antigen presentation, memory CD4^+^ T cell formation is diminished [57]. Our findings indicate that the level of MHC class II antigen presentation during CVB3 infection is sufficient to drive virus-specific memory CD4^+^ T cell differentiation. However, class II antigen presentation may be more limited during CVB3 infection than in LCMV infection, because only half of SMARTA cells in CVB3-infected hosts divided >7 times by day 8 p.i., whereas all of the SMARTA cells in LCMV-infected mice fully diluted their CFSE (Figure 7). Following challenge infection, memory SMARTA cells present in CVB3 immune mice underwent a greater degree of expansion and effector differentiation compared to their counterparts in LCMV immune mice; by day 4, CVB3-induced memory SMARTA cells rapidly and uniformly adopted an effector phenotype (Figure 9D), and a considerable percentage of IFNγ^+^ SMARTA cells lost the capacity to co-produce TNF or IL-2 (Figure 9E & F). In contrast, LCMV-specific memory SMARTA cells became CD127^lo^ more gradually and retained their multi-potential cytokine profile. The more robust secondary SMARTA response in rCVB3.6-immune mice, and the more rapid differentiation of these cells, are most likely attributable to a greater antigen load following LCMV challenge, compared to LCMV-immune mice. This will occur because rCVB3.6-immune mice, unlike their LCMV-immune counterparts, have no detectable GP_33_-specific memory CD8^+^ T cells, and so cannot rapidly control LCMV infection and reduce the antigenic stimulus available to virus-specific CD4^+^ T cells. Another laboratory has recently reported a similar observation, and the authors concluded that the recall response of CD4^+^ memory T cells is, in part, regulated by the duration of the secondary stimulus [58]. The emergence of strong CD8^+^ T cell responses in CVB3-immune mice on day 7 post LCMV challenge coincides temporally with an increase in the proportion of CVB3-specific CD4^+^ T cells that co-produce IFNγ and TNF (Figure 9E & F). Perhaps this CD8^+^ T cell response helps to reduce antigen load, thereby decreasing the frequency of T cell stimulation and permitting SMARTA cells to recover their ability to produce multiple cytokines.

In conclusion, our evaluation of viral antigen presentation with virus-specific CD8^+^ and CD4^+^ transgenic T cells reveals a profound difference in the effect of virus infection on antigen presentation by MHC class I when compared to MHC class II, with a consequent difference in the generation of virus-specific CD8^+^ and CD4^+^ T cell responses. Future efforts to design a CVB3 vaccine should strive to generate high avidity memory CD8^+^ T cells that appear to be more capable than naïve cells of responding to the low level of MHC class I antigen presentation that occurs in CVB3-infected cells [12].

## Materials and Methods

### Mice

C57BL/6J mice were purchased from The Scripps Research Institute (TSRI) breeding facility. P14/Thy1.1 or P14/CD45.1 TCR transgenic mice specific for the H-2D^b^ restricted LCMV epitope GP_33–41_ [59], and SMARTA/CD45.1 TCR transgenic mice specific for the I-A^b^ restricted LCMV epitope GP_61–80_ [60], were bred and maintained by our laboratory as described [25],[61]. All experimental procedures with mice were approved by TSRI Animal Care and Use Committee.

### Viruses and infections

The wtCVB3 used in these studies is a plaque purified isolate (designated H3) of the myocarditic Woodruff variant of CVB3 [62]. Plasmid pH 3, encoding a full-length infectious clone of this virus [63], was provided by Dr. Kirk Knowlton (University of California, San Diego). Five rCVB3, encoding well-characterized T cell epitopes, are used in the experiments described herein. In all cases, the epitopes were inserted immediately downstream of the N-terminus of the CVB3 polyprotein, using the cloning approaches that we have described [11],[12]. All plasmid constructs were confirmed by DNA sequencing. Furthermore, the *in vivo* stability of the dual-epitope insert in rCVB3.6 RNA has been assessed by reverse transcription-PCR, cloning and sequencing at several time points after infection of mice; the dual-epitope sequence remains stable in the recombinant virus for at least 7 days post-infection [12]. The five viruses, and information about the epitope(s) encoded by each, are shown in Table 1. Naïve adult male C57BL/6 mice were inoculated i.p. with 1×10^3^ PFU of wtCVB3, 10^7^–10^8^ PFU of rCVB, or 2×10^5^ PFU of LCMV Armstrong. In some experiments, mice previously infected with CVB3 were inoculated i.p. with 2×10^6^ PFU of LCMV Armstrong.

### Adoptive transfers

The frequency of CD8^+^ transgenic T cells (TCR Vα2^+^Vβ8.1/8.2^+^) in the spleen of male P14/Thy1.1 or P14/CD45.1 mice and the frequency of CD4^+^ transgenic T cells (TCR Vα2^+^Vβ8.3^+^) in the spleen of male SMARTA/CD45.1 mice was determined by flow cytometry. For the majority of experiments, equal numbers of both transgenic cell populations were combined, and a low number (10^4^) of P14 and SMARTA cells [in 0.5 ml DMEM) (Invitrogen, Carlsbad, CA)] was injected i.v. into uninfected adult male C57BL/6J mice. In experiments that analyzed SMARTA T cell responses from different starting precursor frequencies, differing numbers of SMARTA cells (10^1^–10^4^) were transferred. In other experiments that examined transgenic T cell division, P14 and SMARTA transgenic cells were labeled with 5 µM CFSE and a larger number of cells (6–9×10^5^) were transferred into recipient mice. Mice were inoculated with rCVB3, or with LCMV as a positive control, at the time points indicated in the text.

### Plaque assays

To confirm CVB3 infection, the virus titer in the feces was determined on day 2 p.i.. Samples were weighed, disrupted in 0.5 ml DMEM, briefly centrifuged to pellet debris, and the supernatant was used for virus titration. Plaque assays were performed on sub-confluent HeLa cell monolayers as described [64], and the virus titers (PFU/g) were calculated for each sample.

### Lymphocyte isolation

A single cell suspension of splenocytes was prepared by disruption of the spleen through a 70 µm nylon cell strainer (BD Biosciences, San Jose, CA), and red blood cells were lysed with 0.83% NH_4_Cl. In some experiments, mice were anesthetized and perfused with cold PBS, and lymphocytes/mononuclear cells were isolated from the heart and pancreas and purified on a Percoll gradient as described [12]. Lymphocytes isolated from the heart or pancreas were fewer in number, and therefore cells from within each group of mice were pooled prior to flow cytometric analysis. Blood was collected in K_2_EDTA-treated Vacutainer Plus tubes (BD Biosciences) and treated with ACK buffer (0.15 M NH_4_Cl, 1 mM KHCO_3_, and 0.1 mM Na_2_EDTA, pH 7.0) to lyse red blood cells.

### *In vitro* antigen presentation assay

Spleens from uninfected adult male C57BL/6 mice were harvested, finely minced, and digested with collagenase type I (100 U/ml, Worthington Biochemical Corporation, Lakewood, NJ) for 30 min at 37°C, and then filtered through a cell strainer. Splenocytes were resuspended at 10×10^6^ cells/ml in complete RPMI [containing 10% FBS, L-glutamine, 2-mercaptoethanol (50 µM), and penicillin/streptomycin], and incubated with rCVB3 (MOI 10∶1) and/or pulsed with GP_33–41_ peptide (1 µg/ml) for 1 hr at 37°C. Uninfected splenocytes ± GP_33–41_ peptide were prepared in the same manner. Cells were then washed extensively, counted, and 2.5×10^6^ of these stimulator cells were added per well in a 24-well plate. CFSE-labeled transgenic T cells were used as sensors of antigen presentation by these rCVB3-infected stimulator cells. To prepare these indicator cells, splenocytes from uninfected adult male P14/Thy1.1 or SMARTA/CD45.1 TCR transgenic mice were isolated as described above (see Lymphocyte isolation) and the frequencies of CD8^+^ and CD4^+^ transgenic T cells within the spleens were determined by flow cytometry. The P14 and SMARTA splenocytes were labeled with 3 µM CFSE, and were mixed in a ratio that resulted in an equal number of transgenic P14 and SMARTA T cells. Aliquots of this mix were added to the stimulator cells; each well received 3×10^5^ transgenic T cells of each type (P14 and SMARTA). Triplicate cultures were set up for each condition. 72 hours later the *in vitro* cultures were harvested and transgenic T cell proliferation was determined by flow cytometry.

### Intracellular cytokine staining (ICCS)

Prior to intracellular cytokine staining, 1–2×10^6^ splenocytes were incubated for 5 hrs in 96-well plates in 0.2 ml/well RPMI containing 10% FBS, 50 µM 2-mercaptoethanol, penicillin/streptomycin, GolgiPlug or GolgiStop (BD Biosciences), and synthetic peptides (1 µg/ml GP_33–41_, 1 or 10 µM GP_61–80_). After stimulation, the cells were incubated with Fc receptor antibody (Fc Block, BD Biosciences) in PBS containing 2% FBS and 0.1% sodium azide (FACS buffer) for 10 min on ice. Cells were then stained for surface CD4 or CD8, and intracellular IFNγ, TNF, IL-2, IL-4, and/or IL-17A with the Cytofix/Cytoperm kit (BD Biosciences). The total number of (peptide-specific) cytokine-producing T cells was determined by subtracting unstimulated cytokine^+^ T cells from stimulated cytokine^+^ T cells.

### Evaluating T cell activation using phorbol myristate acetate/ionomycin

1–2×10^6^ splenocytes were incubated for 5 hrs with PMA, (50 ng/ml) and ionomycin (500 ng/ml) (both Sigma, St. Louis, MO). Because PMA/ionomycin stimulation causes downregulation of surface CD4 and CD8 [65], intracellular staining was used, as described above, to detect not only IFNγ, but also CD4 and CD8. The total number of cytokine-producing T cells was determined by subtracting unstimulated cytokine^+^ T cells from stimulated cytokine^+^ T cells.

### MHC class II tetramer staining

MHC class II tetramers were provided by the NIH Tetramer Core Facility (Emory University, Atlanta, GA). 1–2×10^6^ splenocytes were incubated for 1.5 or 3 hrs at 37°C in 96-well plates in 0.2 ml/well RPMI containing 2% FBS and allophyocyanin-conjugated I-A^b^/GP_66–77_ tetramers or I-A^b^/hCLIP control tetramers (6 or 18 µg/ml) [23]. Afterwards, the cells were washed and stained for surface CD4, CD8, CD44, and/or CD45.1.

### Flow cytometry materials and analyses

The following materials and analytical methods apply to the ICCS, PMA/ionomycin, and tetramer analyses that have been described above. Fluorochrome-conjugated CD8 (clone 53-6.7), CD4 (clone RM4-5), CD44 (clone IM7), CD45.1 (clone A20), CD62L (clone MEL-14), CD127 (clone A7R34), CD69 (clone H1.2F3), Thy1.1 (CD90.1, clone HIS51), IFN-γ (clone XMG1.2), TNF (clone MP6-XT22), IL-2 (clone JES6-5H4), IL-4 (clone 11B11), and IL-17A (clone eBio17B7) antibodies, and Armenian hamster IgG (eBio299Arm) and rat IgG2a isotype controls, were purchased from eBioscience (San Diego, CA). Vα2 (clone B20.1), Vβ8.1/8.2 (clone MR5-2), Vβ8.3 (clone 1B3.3), CD8 (clone 53-6.7), CD4 (clone RM4-5) antibodies and Mouse BD Fc Block were purchased from BD Biosciences. After staining had been completed, samples were fixed in PBS containing 1% paraformaldehyde and were acquired on a FACS Calibur (BD Biosciences). Data were analyzed with FlowJo software (Tree Star, Ashland, OR).

### Statistical analyses

Statistical significance was determined by an unpaired two-tailed t-test assuming equal variance (Microsoft Excel), or by one way ANOVA with Tukey's post hoc tests (GraphPad Prism). A p value<0.05 was considered statistically significant.
